# Radiofrequency Neurolysis of the Posterior Nasal Nerve: A Systematic Review and Meta‐Analysis

**DOI:** 10.1002/lary.70763

**Published:** 2026-08-02

**Authors:** Eyad Jamileh, Fahad Muhammad, Mohamed Xamza, Khadijah Ahmed, Abdullah Ahmed, Munir Ahmed

**Affiliations:** ^1^ School of Medicine, Queen's University Belfast Belfast UK; ^2^ Royal Blackburn Teaching Hospital; East Lancashire Hospitals Trust Blackburn UK; ^3^ School of Medicine, Queen Mary University of London London UK; ^4^ Department of Medicine University of Sheffield Sheffield UK; ^5^ Department of Diabetes and Endocrinology University Hospitals of Leicester NHS Trust Leicester UK; ^6^ Department of Medicine Southend University Hospital Westcliff‐on‐Sea UK; ^7^ Department of Physics Leyton Sixth Form College London UK

**Keywords:** chronic rhinitis, neurolysis, posterior nasal nerve, radiofrequency ablation, systematic review

## Abstract

**Objectives:**

Temperature‐controlled radiofrequency (TCRF) neurolysis of the posterior nasal nerve is a minimally invasive treatment for chronic rhinitis. While early studies show short‐term efficacy, long‐term durability remains unclear. This study evaluated the short‐ and long‐term efficacy and safety of TCRF neurolysis across discrete follow‐up intervals.

**Data Sources:**

PubMed/MEDLINE, Embase, and the Cochrane Library were searched from inception to March 2026.

**Review Methods:**

Following PRISMA 2020 guidelines (PROSPERO: CRD420261345320), studies evaluating adults undergoing TCRF neurolysis for chronic rhinitis with quantitative outcomes were included. Random‐effects meta‐analyses pooled outcomes at 3 months, 6 months, 12 months, 2 years, and 3 years.

**Results:**

Twelve studies (924 patients) were included. TCRF neurolysis significantly reduced total nasal symptom scores (rTNSS) across all intervals: 3 months (−3.96, 95% CI −4.26 to −3.67), 6 months (−4.50, 95% CI −4.91 to −4.09), 12 months (−4.70, 95% CI −5.04 to −4.37), 2 years (−5.06, 95% CI −5.67 to −4.44), and 3 years (−4.59, 95% CI −5.00 to −4.19). Symptom responder rates (≥ 30% improvement) rose from 74% at 3 months to 81%–84% during long‐term follow‐up. Mini Rhinoconjunctivitis Quality of Life Questionnaire scores demonstrated sustained improvements up to 3 years. Adverse events were infrequent, minor, and transient.

**Conclusion:**

TCRF neurolysis provides safe, clinically meaningful, and durable improvements in nasal symptoms and quality of life for up to 3 years, offering a robust therapeutic option for medically refractory chronic rhinitis.

**Level of Evidence:**

1.

## Graphical abstract

Radiofrequency neurolysis of the posterior nasal nerve significantly improves symptoms of chronic rhinitis, including nasal obstruction and rhinorrhea, while demonstrating a favorable safety profile. This systematic review and meta‐analysis supports the procedure as an effective minimally invasive treatment, although additional high‐quality randomized studies are warranted to further strengthen the evidence. the procedure as an effective minimally invasive treatment, although additional high‐quality randomized studies are warranted to further strengthen the evidence.

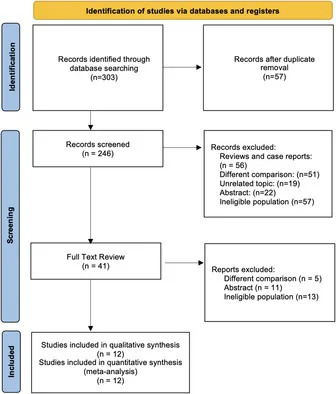

## 1 Introduction

Chronic rhinitis is a common and often persistent condition characterized by symptoms such as rhinorrhea, nasal obstruction, sneezing, and post‐nasal drip [1]. These symptoms can significantly impair sleep, daily functioning, and overall quality of life [2]. Chronic rhinitis may be allergic, non‐allergic, or mixed in etiology, with considerable overlap in clinical presentation. First‐line management typically consists of pharmacological therapy, including intranasal corticosteroids, antihistamines, anticholinergic agents, and allergen immunotherapy [3]. While many patients achieve adequate symptom control, a substantial proportion remain symptomatic despite optimized medical treatment.

For patients with refractory disease, procedural interventions targeting the neural pathways responsible for nasal hypersecretion have been explored. Historically, vidian neurectomy was performed to disrupt parasympathetic innervation of the nasal mucosa, thereby reducing glandular secretion. However, this procedure is technically demanding and associated with notable complications, including dry eye and sensory disturbances, limiting its widespread use [4]. More recently, less invasive techniques have been developed to selectively target the posterior nasal nerve (PNN), which plays a key role in mediating parasympathetic input to the nasal mucosa [5].

Temperature‐controlled radiofrequency (TCRF) neurolysis of the PNN has emerged as a minimally invasive, office‐based intervention for patients with medically refractory chronic rhinitis. By delivering controlled thermal energy to the PNN region, this technique aims to reduce parasympathetic activity and, consequently, nasal hypersecretion and congestion [6]. Early clinical studies have demonstrated promising improvements in symptom burden and quality of life, with a favorable safety profile [7].

Previous systematic reviews have evaluated the efficacy of PNN‐targeted interventions; however, these analyses have largely focused on short‐term outcomes or pooled follow‐up data without detailed assessment of symptom durability over time [8, 9]. In particular, there remains a lack of comprehensive synthesis examining the trajectory of treatment effects across discrete follow‐up intervals extending beyond one year. Understanding the long‐term sustainability of symptom improvement is essential for informing clinical decision‐making and patient counseling.

Therefore, this systematic review and meta‐analysis aimed to evaluate the efficacy and safety of TCRF neurolysis of the PNN in patients with chronic rhinitis. Specifically, we performed a time‐stratified analysis of symptom outcomes and quality‐of‐life measures across multiple follow‐up intervals, in order to assess both the magnitude and durability of treatment effects.

## 2 Methods

### 2.1 Study Design and Registration

This systematic review and meta‐analysis was reported in accordance with the Preferred Reporting Items for Systematic Reviews and Meta‐Analyses (PRISMA 2020) guidelines [10]. The study protocol was prospectively registered on PROSPERO (PROSPERO ID: CRD420261345320) [11].

### 2.2 Search Strategy

A comprehensive literature search was performed across PubMed/MEDLINE, Embase, and the Cochrane Library from database inception to March 2026. The search strategy incorporated a combination of controlled vocabulary (MeSH and Emtree terms) and free‐text keywords related to chronic rhinitis, PNN, and radiofrequency neurolysis. The full search strategy is provided in Supporting Information. In addition, the reference lists of included studies and relevant systematic reviews were manually screened to identify any additional eligible studies.

### 2.3 Eligibility Criteria

Studies were included if they evaluated adult patients with chronic rhinitis (allergic, non‐allergic, or mixed) undergoing TCRF neurolysis of the PNN and reported quantitative outcomes such as symptom scores, quality‐of‐life measures, or responder rates. Eligible study designs included randomized controlled trials (RCTs) as well as prospective and retrospective cohort studies. Studies were excluded if they were case reports, reviews, editorials, or conference abstracts without full data, included fewer than 10 patients, or did not provide extractable quantitative outcome data.

### 2.4 Study Selection

Study selection was performed independently by two reviewers. Titles and abstracts were screened for eligibility, followed by full‐text assessment of potentially relevant studies. Any discrepancies were resolved through discussion, with the involvement of a third reviewer where necessary to reach consensus.

### 2.5 Data Extraction

Data extraction was conducted independently by two reviewers using a standardized data collection form. Extracted variables included study characteristics (author, year, design, and sample size), patient demographics and rhinitis subtype, intervention details, follow‐up duration, and reported outcomes. Outcome data included total nasal symptom scores (rTNSS or TNSS), quality‐of‐life measures such as the mini‐RQLQ, responder rates, and adverse events. Where necessary, missing or incomplete data were derived from reported statistics, including confidence intervals (CI), standard errors, *p*‐values, or graphical representations.

### 2.6 Outcomes

The primary outcome was the change in total nasal symptom score (rTNSS or TNSS) from baseline at predefined follow‐up intervals. Secondary outcomes included responder rates, quality‐of‐life measures, and safety outcomes, including adverse events and complications. A time‐stratified approach was adopted, with outcomes analyzed separately at 3 months, 6 months, 12 months, 2 years, and 3 years, rather than pooling across heterogeneous follow‐up periods.

### 2.7 Data Synthesis and Statistical Analysis

Meta‐analysis was performed using a random‐effects model to account for anticipated clinical and methodological heterogeneity across studies. Continuous outcomes were pooled as mean differences with corresponding 95% CI using inverse‐variance weighting. Where change scores were not directly reported, they were calculated from baseline and follow‐up means. Standard deviations were derived from available summary statistics, including CI, standard errors, or *p*‐values, using established statistical methods where required. Proportion outcomes, such as responder rates, were pooled using the Freeman‐Tukey double arcsine transformation to stabilize variance, with results presented as pooled proportions and 95% CI. All analyses were conducted using Meta‐Analysis.com which applied appropriate transformations and inverse‐variance weighting for meta‐analytic calculations [12]. Statistical heterogeneity was assessed using the I^2^ statistic, with values of 25%, 50%, and 75% representing low, moderate, and high heterogeneity, respectively. Sensitivity analyses were limited by the small number of included studies per outcome; therefore, no formal sensitivity analyses were undertaken. These limitations were considered when interpreting the robustness of the pooled estimates.

### 2.8 Risk of Bias Assessment

The methodological quality of included studies was assessed independently by two reviewers. RCTs were evaluated using the Cochrane Risk of Bias 2 (RoB 2) tool, while non‐randomized studies were assessed using the National Institute of Health (NIH) Quality Assessment Tool [13, 14]. Disagreements were resolved through discussion and consensus. Publication bias could not be formally assessed due to the limited number of studies available for each outcome, as fewer than 10 studies contributed to individual meta‐analyses. Consequently, funnel plot assessment and formal statistical testing using Egger's regression were not performed.

## 3 Results

### 3.1 Literature Search Results

The literature search identified 303 records. After removal of duplicates and screening of titles and abstracts, full‐text assessment was performed for potentially eligible studies. A total of 12 studies met the inclusion criteria and were included in the final analysis (Figure 1).

**FIGURE 1:**
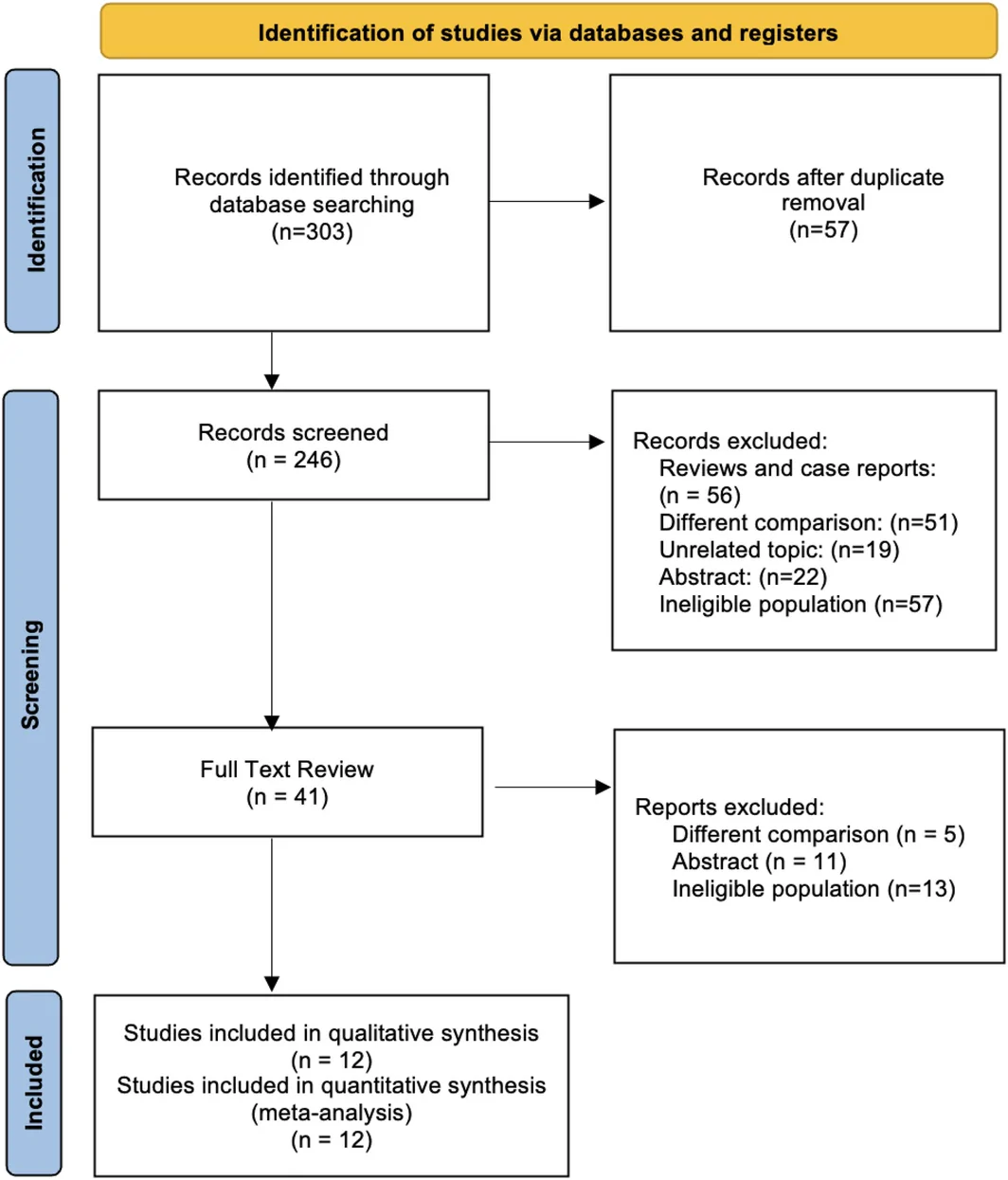
PRISMA diagram. [Color figure can be viewed in the online issue, which is available at www.laryngoscope.com]

### 3.2 Description of Studies

A total of 12 studies comprising 924 patients were included in the final analysis. All studies evaluated adults with chronic rhinitis undergoing TCRF neurolysis of the PNN using either the RhinAer or NEUROMARK systems [6, 7, 15, 16, 17, 18, 19, 20, 21, 22, 23, 24, 25]. The included studies consisted of a combination of RCTs and prospective cohort studies, including several extension studies reporting longer‐term follow‐up. Three studies were sham‐controlled RCTs, while the remainder were prospective single‐arm cohorts. Follow‐up durations ranged from 3 months to 3 years, allowing for both short‐ and long‐term assessment of outcomes. Across studies, participants had a mean age of approximately 57–63 years, with a slight predominance of female patients. The study populations included patients with allergic, non‐allergic, and mixed rhinitis, with non‐allergic rhinitis being the most reported subtype. Many patients had a history of prior nasal surgery and were receiving baseline medical therapy, including antihistamines and intranasal corticosteroids. Baseline characteristics of the included studies are summarized in Table 1, and detailed participant characteristics are presented in Table S1. All studies reported changes in total nasal symptom scores (rTNSS or TNSS), with several also reporting responder rates, quality‐of‐life outcomes (mini‐RQLQ), and safety data across multiple follow‐up intervals. A summary of the reported outcomes is presented in Table S2.

**TABLE 1:** Baseline characteristics of the studies included.

Author (year)	Journal	Region	Study design	Single‐centre or multi‐centre	Follow‐up duration	Total sample (*n*)	Population	Comparison	Device
Ehmer et al. (2022) [6]	American Journal of Rhinology & Allergy	USA	Prospective (single‐arm)	Single	12 months	50	Chronic rhinitis	Pre‐post	RhinAer system
Ehmer et al. (extension) [12]	American Journal of Rhinology & Allergy	USA	Prospective (extension)	Single	24 months	34	Chronic rhinitis	Pre‐post	RhinAer system
Lee et al. (2022) [13]	American Journal of Rhinology & Allergy	USA	Prospective (single‐arm)	Multi	6 months	129	Chronic rhinitis	Pre‐post	RhinAer system
Lee et al. (2025) (extension) [14]	American Journal of Rhinology & Allergy	USA	Prospective (ext)	Multi	36 months	101	Chronic rhinitis	Pre‐post	RhinAer system
Stolovitzky et al. (2021) [7]	American Academy of Otolaryngology–Head and Neck Surgery	USA	RCT	Multi	3 months	116	Chronic rhinitis	Sham	RhinAer system
Takashima et al. (2022) (extension) [15]	International Forum of Allergy & Rhinology	USA	RCT (extension)	Multi	12 months	104	Chronic rhinitis	Crossover	RhinAer system
Takashima et al. (2024) (extension) [16]	International Forum of Allergy & Rhinology	USA	RCT (extension)	Multi	24 months	79	Chronic rhinitis	Crossover	RhinAer system
Stolovitzky et al. (2025) (extension) [17]	International Forum of Allergy & Rhinology	USA	RCT (extension)	Multi	36 months	59	Chronic rhinitis	Crossover	RhinAer system
Reh et al. (2023) [18]	Laryngoscope Investigative Otolaryngology	USA	Prospective (single‐arm)	Multi	3 months	36	Chronic rhinitis	Pre‐post	NEUROMARK system
Reh et al. (2024) (extension) [19]	Laryngoscope Investigative Otolaryngology	USA	Prospective (extension)	Multi	12 months	35	Chronic rhinitis	Pre‐post	NEUROMARK system
Davis et al. (2024) [20]	Ear, Nose & Throat Journal	USA	Prospective (single‐arm)	Multi	6 months	80	Chronic rhinitis	Pre‐post	NEUROMARK system
Yen et al. (2025) (extension) [21]	Ear, Nose & Throat Journal	USA	Prospective (extension)	Multi	24 months	75	Chronic rhinitis	Pre‐post	NEUROMARK system
Takashima et al. (2026) [22]	International Forum of Allergy & Rhinology	USA	RCT	Multi	3 months	125	Chronic rhinitis	Sham	NEUROMARK system

### 3.3 Change in rTNSS

A total of four studies (*n* = 325) were included in the analysis of rTNSS at 3 months [7, 16, 21, 25] The pooled mean change was −3.96 (95% CI −4.26 to −3.67), with no observed heterogeneity (I^2^ = 0%). At 6 months, three studies (*n* = 272) [16, 18, 23] demonstrated a greater reduction in symptom burden, with a pooled mean change of −4.50 (95% CI −4.91 to −4.09) and low‐to‐moderate heterogeneity (I^2^ = 28.6%). At 12 months, four studies (*n* = 312) [6, 14, 15, 20] showed sustained improvement, with a pooled mean change of −4.70 (95% CI −5.04 to −4.37) and low heterogeneity (I^2^ = 19.7%). Long‐term follow‐up demonstrated continued benefit. At 2 years (three studies, *n* = 220) [6, 17, 19], the pooled mean change was −5.06 (95% CI −5.67 to −4.44), with substantial heterogeneity (I^2^ = 65.4%). At 3 years (two studies, *n* = 160) [17, 20], the pooled mean change was −4.59 (95% CI −5.00 to −4.19), with no heterogeneity (I^2^ = 0%). Overall, symptom improvement was evident as early as 3 months, increased through 12 months, and remained durable up to 3 years, with the greatest magnitude of effect observed at 2 years. This is represented in Figure 2.

**FIGURE 2:**
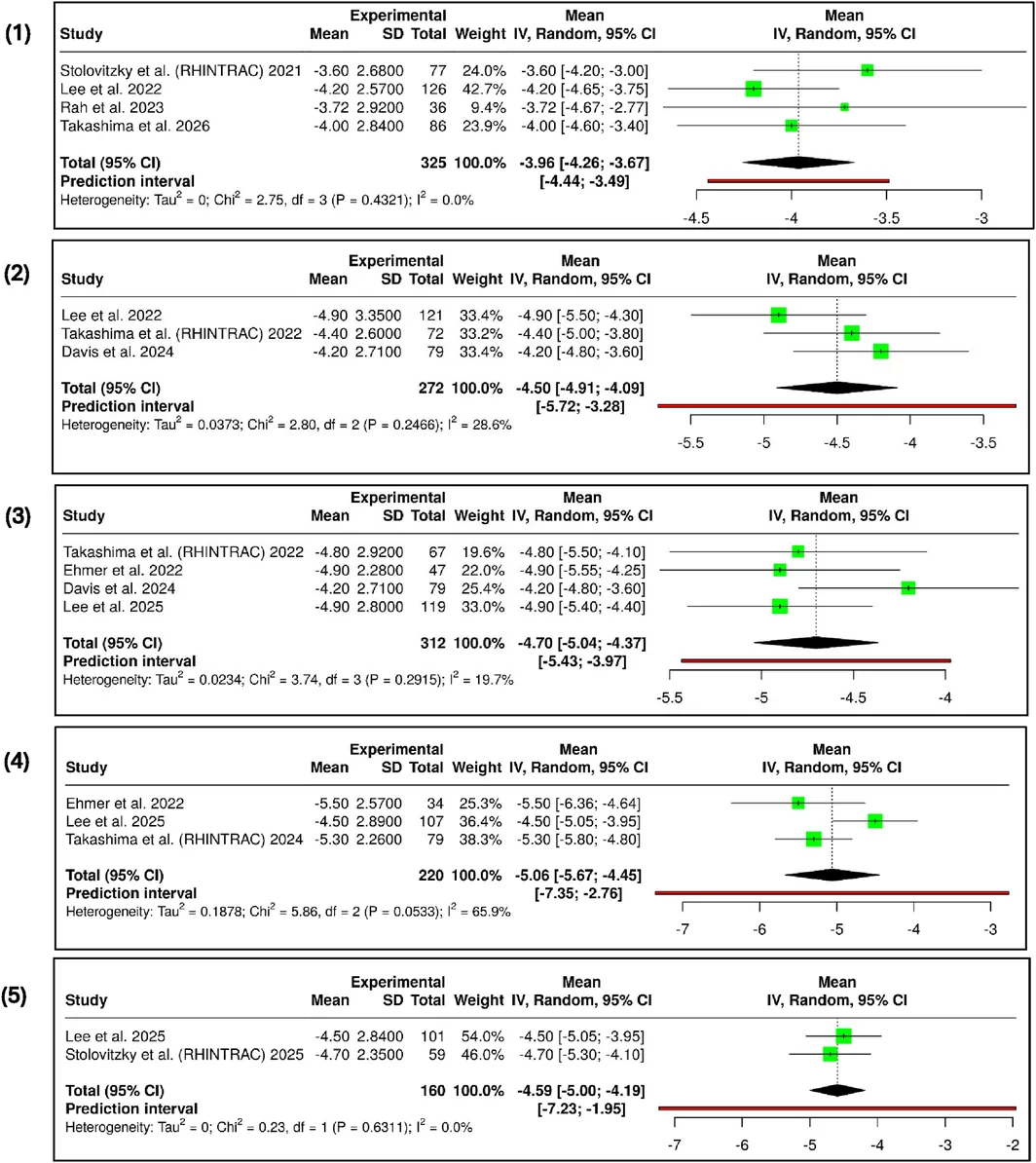
Forest plots showing the pooled mean change in reflective Total Nasal Symptom Score (rTNSS) at (1) 3 months, (2) 6 months, (3) 12 months, (4) 2 years, and (5) 3 years follow‐up. [Color figure can be viewed in the online issue, which is available at www.laryngoscope.com]

### 3.4 Responder Rates (≥ 30% Improvement in rTNSS)

Responder rates increased over time and remained consistently high across all follow‐up intervals. At 3 months (four studies, *n* = 325) [7, 13, 18, 22], the pooled responder rate was 74% (95% CI 69%–78%; I^2^ = 0%). At 6 months (four studies, *n* = 320) [6, 16, 18, 23], the responder rate increased to 82% (95% CI 75%–88%), with moderate heterogeneity (I^2^ = 56.5%). At 12 months (four studies, *n* = 268) [6, 17, 18, 22], the pooled responder rate was 84% (95% CI 79%–89%), with minimal heterogeneity (I^2^ = 6.3%). At 2 years (three studies, *n* = 220) [12, 17, 19], the responder rate remained high at 84% (95% CI 76%–90%; I^2^ = 44.1%). At 3 years (two studies, *n* = 160) [17, 20], the pooled responder rate was 81% (95% CI 75%–87%; I^2^ = 0%). These findings demonstrate a sustained and clinically meaningful treatment response in the majority of patients over long‐term follow‐up. This is represented in Figure 3.

**FIGURE 3:**
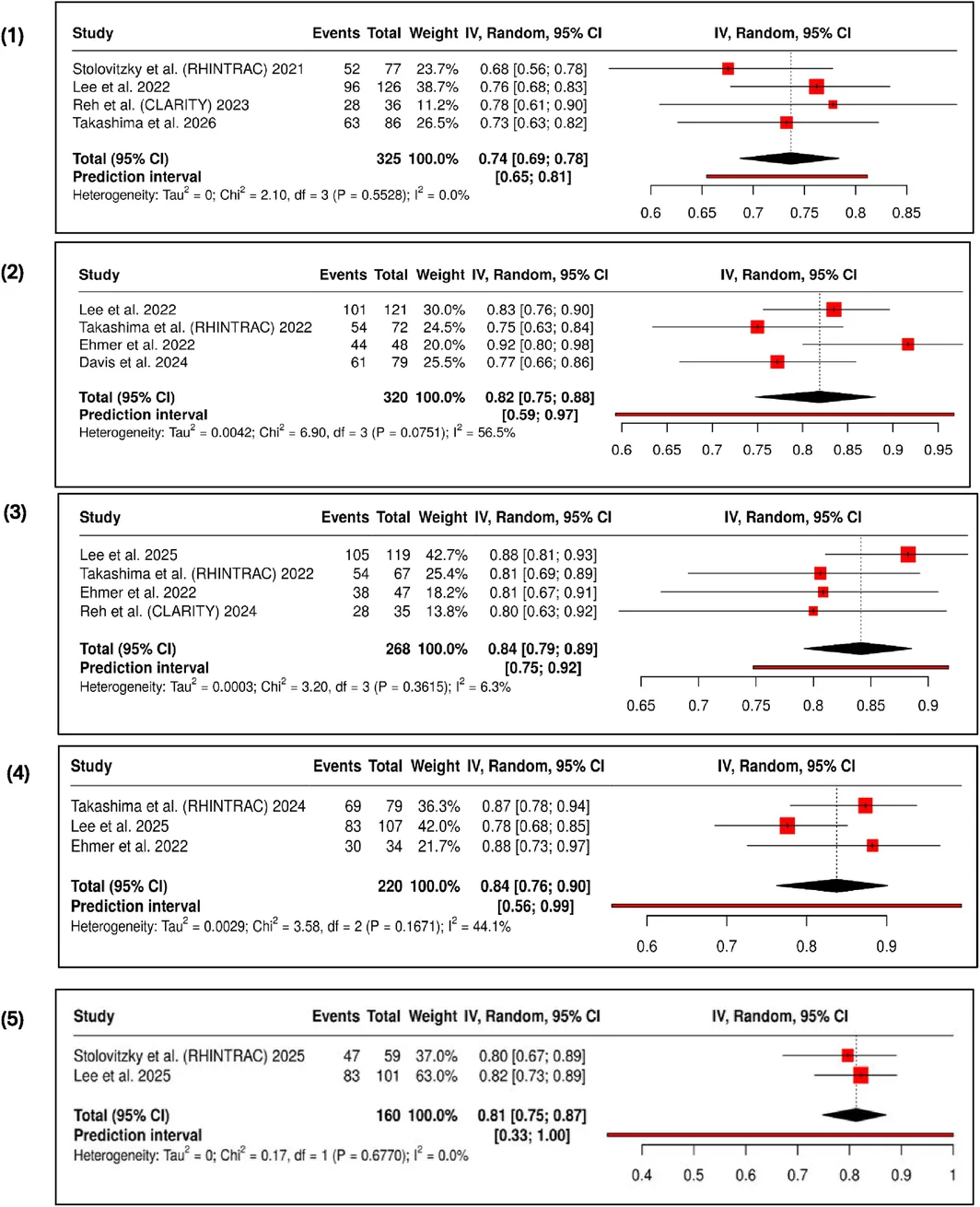
Forest plots showing the pooled responder rates (≥ 30% improvement in reflective Total Nasal Symptom Score [rTNSS]) at (1) 3‐months, (2) 6‐months, (3) 12‐months, (4) 2‐years, and (5) 3‐years follow‐up. [Color figure can be viewed in the online issue, which is available at www.laryngoscope.com]

### 3.5 Quality of Life (Mini‐RQLQ)

Significant improvements in quality of life were observed across all time points. At 3 months (three studies, *n* = 291) [16, 23, 25], the pooled mean change in mini‐RQLQ was −1.70 (95% CI −1.87 to −1.53; I^2^ = 0%). At 6 months (two studies, *n* = 200) [13, 20], the pooled mean change remained consistent at −1.70 (95% CI −1.91 to −1.50; I^2^ = 0%). At 3 years (two studies, *n* = 160) [17, 20], improvement was sustained with a pooled mean change of −1.80 (95% CI −1.98 to −1.62; I^2^ = 0%). Across studies, a high proportion of patients achieved the minimum clinically important difference (MCID ≥ 0.4), exceeding 80% at early follow‐up and remaining durable at long‐term assessment. This is represented in Figure 4.

**FIGURE 4:**
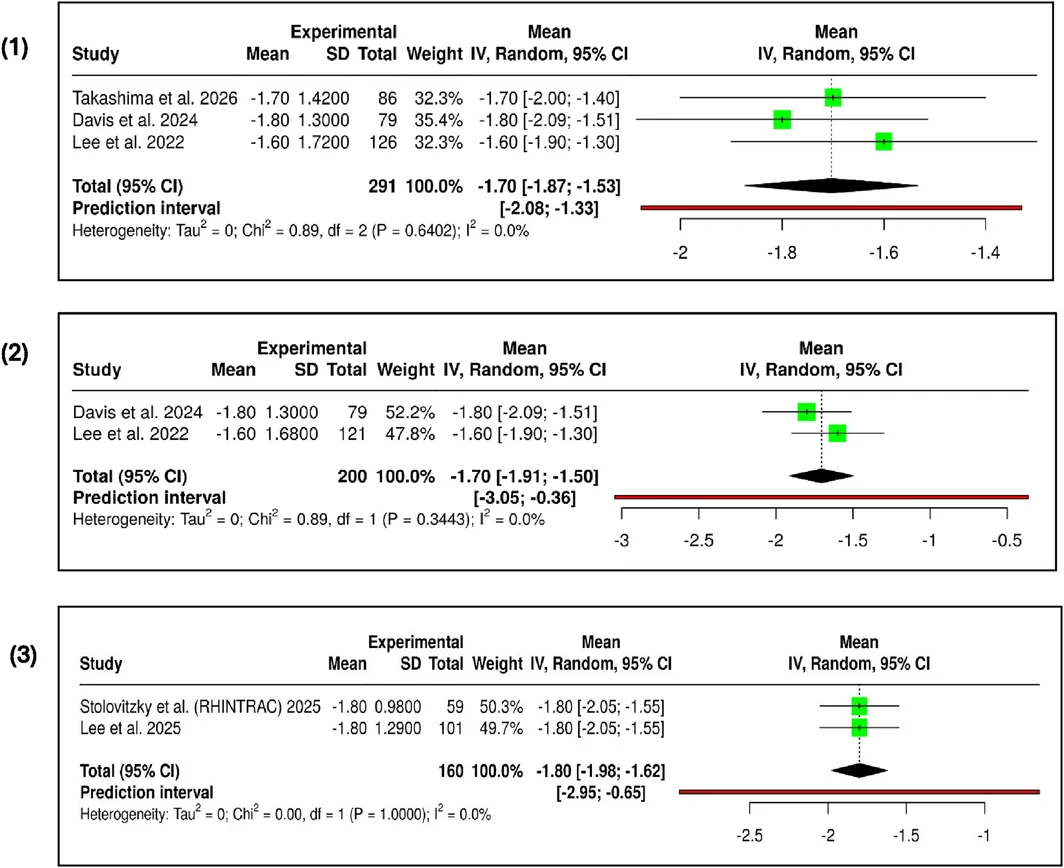
Forest plots showing the pooled mean change in Mini Rhinoconjunctivitis Quality of Life Questionnaire (Mini‐RQLQ) scores at (1) 3‐months, (2) 6‐months, and (3) 3‐years follow‐up. [Color figure can be viewed in the online issue, which is available at www.laryngoscope.com]

### 3.6 Symptom Subscores

Improvements were observed across all individual rTNSS components, including rhinorrhoea, nasal congestion, sneezing, itching, and postnasal drip. Reductions in rhinorrhoea and congestion were consistently the most pronounced contributors to overall symptom improvement, particularly in the RHINTRAC and NEUROMARK cohorts [7, 18, 19, 20, 21, 22, 23, 24, 25]. Postnasal drip and cough scores also improved and were sustained up to 3 years in long‐term follow‐up studies [17, 20].

### 3.7 Additional Patient‐Reported Outcomes

Where reported, Nasal Obstruction Symptom Evaluation (NOSE) scores demonstrated substantial improvement, with reductions of approximately −30 to −36 points within the first 6 months [23, 24, 25]. Other outcomes, including Eustachian Tube Dysfunction Questionnaire‐7 (ETDQ‐7) scores [23, 24], also improved, suggesting potential benefits in Eustachian tube dysfunction. One RCT reported improvement in depression scores (Patient Health Questionnaire‐9), with greater reductions observed in the treatment group compared to sham [25].

### 3.8 Medication Use

Several studies reported a reduction in medication burden following treatment. Patients were more likely to reduce or discontinue intranasal therapies, antihistamines, and oral medications from 6 months onwards, with sustained reductions observed at 2‐ and 3‐year follow‐up [6, 17, 19].

### 3.9 Safety and Tolerability

TCRF neurolysis demonstrated a favorable safety profile across all included studies. Procedure‐related pain was generally mild and transient, with no reports of persistent or clinically significant long‐term nasal pain. No serious device‐ or procedure‐related adverse events were reported in most studies, including those with long‐term follow‐up. Non‐serious adverse events were infrequent and included transient epistaxis, nasal discomfort, and minor procedural symptoms. One study reported a serious adverse event (epistaxis requiring hospitalization and intervention), which was rare [23]. In RCTs, adverse event rates were comparable between treatment and sham groups, with no new safety concerns identified over long‐term follow‐up.

### 3.10 Risk of Bias

Among randomized studies, the overall risk of bias was largely high, driven by concerns in deviations from intended interventions, missing outcome data, and outcome measurement. In contrast, bias from randomization and selective reporting was consistently low. For non‐randomized studies, most were rated as good quality (6/8), with two fair‐quality studies. Key limitations included a lack of blinding across all studies and inconsistent reporting of sample size justification. Overall, the evidence is limited by a high risk of bias in randomized studies and design‐related limitations in pre‐post studies. These are summarized in Figure 5 and Table 2.

**FIGURE 5:**
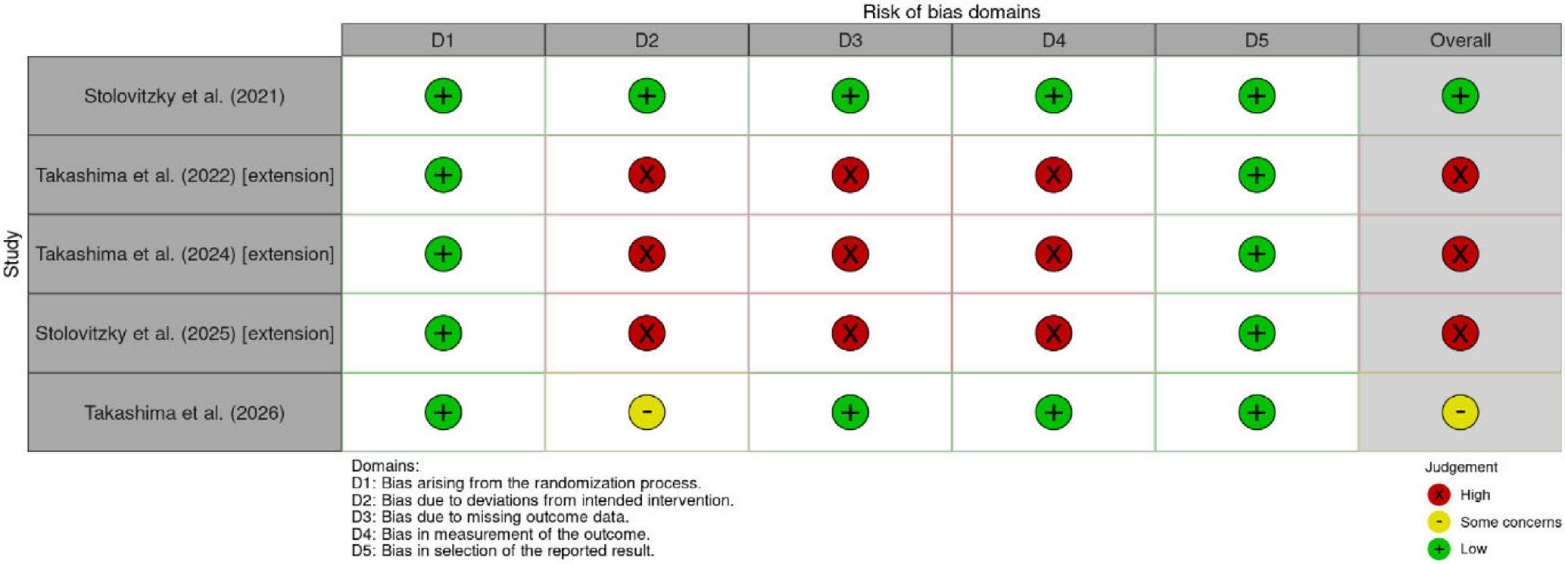
Risk of bias of randomized studies presented via a traffic light plot using the Cochrane Risk of Bias‐2 tool. [Color figure can be viewed in the online issue, which is available at www.laryngoscope.com]

**TABLE 2:** Risk of bias summary for each individual study using the National Institutes of Health (NIH) quality assessment tool for before‐after (pre‐post) studies with no control group.

Study	Q1	Q2	Q3	Q4	Q5	Q6	Q7	Q8	Q9	Q10	Q11	Q12	Overall Quality Rating
Ehmer et al., 2022 (12 months)	Y	Y	Y	CD	Y	Y	Y	N	Y	Y	Y	NA	Good
Ehmer et al., 2022 (24 months)	Y	Y	Y	N	N	Y	Y	N	N	Y	Y	NA	Fair
Lee et al., 2022 (6 months)	Y	Y	Y	CD	N	Y	Y	N	Y	Y	Y	NA	Good
Lee et al., 2025 (3 years)	Y	Y	Y	CD	Y	Y	Y	N	N	Y	Y	NA	Fair
Reh et al., 2023 (3 months)	Y	Y	Y	CD	Y	Y	Y	N	Y	Y	Y	NA	Good
Reh et al., 2024 (12 months)	Y	Y	Y	CD	N	Y	Y	N	Y	Y	Y	NA	Good
Davis et al., 2024 (6 months)	Y	Y	Y	CD	Y	Y	Y	N	Y	Y	Y	NA	Good
Yen et al., 2025 (24 months)	Y	Y	Y	CD	N	Y	Y	N	Y	Y	Y	NA	Good

*Note:* Y = Yes; N = No; CD = Cannot Determine; NA = Not Applicable.

## 4 Discussion

This systematic review and meta‐analysis demonstrate that TCRF neurolysis of the PNN is associated with significant, clinically meaningful, and durable improvements in both nasal symptom burden and quality of life in patients with chronic rhinitis. To our knowledge, this represents the first meta‐analysis to comprehensively evaluate long‐term outcomes extending to 3 years, addressing a key gap in the current literature. However, the findings of this meta‐analysis should be interpreted with caution, as several outcomes were based on a limited number of included studies.

Across all analyzed time points, TCRF was associated with a consistent reduction in rTNSS, with improvements evident as early as 3 months and sustained throughout long‐term follow‐up. Notably, the magnitude of improvement increased over time, peaking at approximately 2 years before stabilizing at 3 years. This trajectory suggests that the therapeutic effect of TCRF is not transient, but rather reflects a durable modification of underlying disease mechanisms, potentially mediated by sustained attenuation of parasympathetic neural signaling [6, 8].

The biological plausibility of these findings is well supported by the mechanism of action of TCRF. The PNN provides parasympathetic innervation to the nasal mucosa, regulating glandular secretion and vascular tone [26]. Selective neurolysis of this pathway reduces mucosal hypersecretion and vascular engorgement—key drivers of rhinorrhoea and nasal congestion in both allergic and non‐allergic rhinitis [27]. In contrast to vidian neurectomy, which interrupts parasympathetic fibers more proximally and carries risks such as xerophthalmia and sensory disturbance, TCRF offers a targeted, minimally invasive approach that preserves surrounding neural structures while achieving meaningful symptom control [8].

Responder analyses further reinforce the clinical significance of these findings. A substantial proportion of patients achieved a ≥ 30% reduction in rTNSS across all follow‐up intervals, with responder rates increasing from 74% at 3 months to approximately 80%–84% at longer‐term follow‐up. This progressive increase in responder rates may reflect ongoing neural adaptation or sustained suppression of parasympathetic activity, suggesting that the benefits of TCRF may consolidate over time rather than diminish.

Improvements in symptom burden were paralleled by significant gains in quality of life. Reductions in mini‐RQLQ scores consistently exceeded the established MCID, indicating that the observed changes are not only statistically significant but also meaningful from the patient perspective. Importantly, these benefits were sustained at long‐term follow‐up, supporting the durability of treatment effect. Furthermore, reductions in medication use observed across multiple studies suggest that TCRF may decrease reliance on long‐term pharmacotherapy, which has important implications for patient adherence, side‐effect burden, and healthcare utilization [6, 7, 15, 16, 17, 18, 19, 20].

Analysis of individual symptom domains demonstrated consistent improvement across all components of the rTNSS, including rhinorrhea, nasal congestion, sneezing, itching, and postnasal drip. The most pronounced improvements were observed in rhinorrhea and congestion, which aligns with the known physiological role of parasympathetic innervation in nasal glandular secretion and vascular regulation. Improvements in additional patient‐reported outcomes, including NOSE and ETDQ‐7 scores, further support the broad and clinically relevant impact of the intervention beyond traditional rhinitis metrics.

TCRF neurolysis demonstrated a favorable safety profile across all included studies. Serious device‐ or procedure‐related adverse events were rare, with only a single reported case of significant epistaxis requiring intervention [23, 24]. Non‐serious adverse events were infrequent and generally mild and self‐limiting. Importantly, no new safety concerns emerged with extended follow‐up up to 3 years. These findings compare favorably with more invasive surgical approaches and support the role of TCRF as a safe, office‐based intervention for patients with refractory symptoms.

Importantly, these findings are not only statistically significant but also clinically meaningful. The magnitude of improvement in rTNSS exceeded the minimal clinically important difference reported for chronic rhinitis, while improvements in Mini‐RQLQ consistently surpassed the established MCID. Furthermore, the sustained responder rates and reductions in medication use suggest that these benefits translate into meaningful improvements in daily symptom control, quality of life, and treatment burden for patients with medically refractory chronic rhinitis.

Heterogeneity across analyses was generally low, particularly for primary and quality‐of‐life outcomes, indicating consistent treatment effects across diverse study populations and settings. The moderate heterogeneity observed in the 2‐year analysis likely reflects differences in study design, patient selection, and follow‐up completeness; however, the direction and magnitude of effect remained consistent, supporting the robustness of the overall findings.

Despite these strengths, several limitations warrant consideration. The majority of included studies were single‐arm prospective cohorts, which introduces the potential for selection bias and limits causal inference. Although sham‐controlled randomized trials were included and demonstrated superior outcomes with active treatment, the overall number of high‐quality comparative studies remains limited. Additionally, variability in outcome reporting and follow‐up intervals restricted the ability to perform more comprehensive pooled analyses for certain secondary outcomes. Incomplete long‐term follow‐up in some cohorts may also introduce attrition bias. Furthermore, several meta‐analyses included only a small number of studies. Consequently, the pooled estimates may be less precise and more susceptible to the influence of individual studies. Therefore, these findings should be interpreted cautiously until larger, high‐quality comparative studies become available. Also, most of the studies originated from the United States, which may limit the generalisability of the findings to other healthcare settings and populations.

The potential influence of the placebo effect must also be considered, particularly in non‐controlled studies. However, the presence of sham‐controlled trials demonstrating significantly greater improvements in the active treatment groups supports a true treatment effect beyond placebo. Moreover, the durability of benefit observed at 2‐ and 3‐year follow‐up further argues against a purely placebo‐driven response.

Future research should focus on head‐to‐head comparative trials evaluating TCRF against other PNN interventions, including cryotherapy and laser ablation, to better define relative efficacy, durability, and cost‐effectiveness. There is also a need for studies identifying predictors of treatment response, including rhinitis subtype, baseline symptom severity, and comorbid conditions. Standardization of outcome reporting, particularly for symptom subscores and patient‐reported measures, would enhance comparability and facilitate more robust meta‐analytic synthesis.

## 5 Conclusion

TCRF neurolysis of the PNN is an effective, safe, and durable treatment for chronic rhinitis, resulting in significant improvements in nasal symptoms and quality of life that are sustained for up to 3 years. With high responder rates, a favorable safety profile, and the potential to reduce long‐term medication use, TCRF represents a valuable minimally invasive therapeutic option for patients with persistent symptoms despite optimal medical management.

## Funding

The authors have nothing to report.

## Ethics Statement

The authors have nothing to report.

## Conflicts of Interest

The authors declare no conflicts of interest.

## Supporting information


**Data S1:** PRISMA 2020 checklist.

**Data S2:** PubMed/MEDLINE search strategy.

**Table S1:** Baseline characteristics of participants included.
**Table S2:** Outcomes from the studies included.

## Data Availability

The data that support the findings of this study are available from the corresponding author upon reasonable request.
