# Supplementary material for: Radiofrequency Neurolysis of the Posterior Nasal Nerve: A Systematic Review and Meta‐Analysis
Source: Laryngoscope. 2026 Aug 2;136(12):5199–210. doi: 10.1002/lary.70763 (PMC13765444; doi:10.1002/lary.70763)
Supplement: Supplementary file 1 [file LARY-136-5199-s001.docx]

# PubMed/MEDLINE Search Strategy

("Rhinitis"[Mesh] OR rhinitis OR "chronic rhinitis" OR "allergic rhinitis" OR "non-allergic rhinitis")
AND
("Posterior Nasal Nerve" OR PNN OR "posterior nasal nerve")
AND
("Radiofrequency Ablation" OR "radiofrequency neurolysis" OR "radiofrequency" OR TCRF OR RhinAer OR NEUROMARK)

# Embase Search Strategy

('rhinitis'/exp OR rhinitis OR 'chronic rhinitis' OR 'allergic rhinitis' OR 'non allergic rhinitis')
AND
('posterior nasal nerve' OR pnn)
AND
('radiofrequency ablation'/exp OR 'radiofrequency neurolysis' OR 'radiofrequency' OR tcrf OR rhinaer OR neuromark)

# Cochrane Library Search Strategy

(rhinitis OR "chronic rhinitis" OR "allergic rhinitis" OR "non-allergic rhinitis")
AND
("posterior nasal nerve" OR PNN)
AND
("radiofrequency ablation" OR "radiofrequency neurolysis" OR TCRF OR RhinAer OR NEUROMARK)
