# Supplementary material for: Radiofrequency Neurolysis of the Posterior Nasal Nerve: A Systematic Review and Meta‐Analysis
Source: Laryngoscope. 2026 Aug 2;136(12):5199–210. doi: 10.1002/lary.70763 (PMC13765444; doi:10.1002/lary.70763)
Supplement: Supplementary file 2 [file LARY-136-5199-s002.docx]

**Radiofrequency Neurolysis of the Posterior Nasal Nerve: A Systematic Review and Meta-analysis**

*Supplementary Materials*

**Table 1: Baseline characteristics of participants included.**

| **Characteristic** | **Ehmer et al. (2022)**  **[6]** | Ehmer et al. (ext) [12] | **Lee et al. (2022) [13]** | Lee et al. (2025) (ext) [14] | **Stolovitzky et al. (2021) [7]** | Takashima et al.(2022) (ext [15] | Takashima et al. (2024) (ext) [16] | Stolovitzky et al. (2025) (ext) [17] | Reh et al. (2023)  [18] | Reh et al. (2024) (ext) [19] | Davis et al. (2024) [20] | Yen et al. (2025) (ext) [21] | Takashima et al. (2026)  [22] |
| --- | --- | --- | --- | --- | --- | --- | --- | --- | --- | --- | --- | --- | --- |
| Baseline n | 50 | 34 | 129 | 101 | 116 | 77 | 79 | 59 | 36 | 35 | 80 | 75 | 86 |
| Age, years (SD) | 57.9 (11.9) | 57.9 (11.9) | 57.9 (13.4) | 58 (13.6) | 57.3 (14.8) | 57.3 ± 14.8 | 57.9 (13.3) | 56.5 (13.2) | 62.8 (14.0) | 63.2 (14.0) | 59.2 (15.8) | 60.9 (14.6) | 59.4 (14.7) |
| Female, n (%) | 29 (58.0) | 21 (62.0) | 69 (53.5) | 57 (56.4) | 49 (63.6) | 49 (63.6) | 48 (61.0) | 36 (61.0) | 19 (52.8) | 19 (54.3) | 29 (36.2) | 46 (61.3) | 34 (39.5) |
| Race, n (%) |  |  |  |  |  |  |  |  |  |  |  |  |  |
| White | 47 (94.0) | 21 (94.0) | 117 (90.7) | 90 (89.0) | 69 (89.6) | 69 (89.6) | 73 (92.0) | 54 (91.5) | 32 (88.9) | 31 (88.6) | 70 (87.5) | 65 (86.7) | 72 (83.7) |
| Black | 0 (0) | 0 (0) | 5 (3.9) | 5 (5.0) | 5 (6.5) | 5 (6.5) | 4 (5.1) | 3 (5.1) | 3 (8.3) | 3 (8.6) | 8 (10.0) | 8 (10.7) | 2 (2.3) |
| Asian | 2 (4.0) | 1 (3.0) | 4 (3.1) | 4 (4.0) | 1 (1.3) | 1 (1.3) | 2 (2.5) | 2 (3.4) | 1 (2.8) | 1 (2.9) | 1 (1.2) | 1 (1.3) | 7 (8.1) |
| Other | 1 (2.0) | 1 (3.0) | 3 (2.3) | 2 (2.0) | 2 (2.6) |  |  | 0 (0.0) |  |  | 1 (1.2) | 1 (1.3) | 5 (5.8) |
| Rhinitis Subtype |  |  |  |  |  |  |  |  |  |  |  |  |  |
| Allergic | 21 (42.0) | 12 (38.0) | 10 (7.8) | 8 (8.0) | NR | NR | 8 | 3 (5.1) | NR | NR | 27 (33.8) | 26 (34.7) | NR |
| Non-allergic | 17 (34.0) | 10 (29.0) | 93 (72.1) | 73 (73.0) | NR | NR | 32 | 25 (42.4) | NR | NR | 48 (60.0) | 45 (60.0) | NR |
| Mixed | NR | NR | 1 (0.8) | 1 (1.0) | NR | NR | 1 | 0 (0.0) | NR | NR | 5 (6.2) | 4 (5.3) | NR |
| Other | 12 (24.0) | 11 (32.0) | 25 (19.4) | 19 (19.0) | NR | NR | 38 | 31 (52.5) | NR | NR | NR | NR | NR |
| Prior Nasal Surgery | NR | NR | 44 (34.1) | 28 (28.0) | 27 (35.1) | 27 (35.1) | 29 (36.7) | 21 (35.6) | 15 (41.7) | 14 (40.0) | 28 (35.0) | NR | 33 (38.4) |
| Baseline Medication |  |  |  |  |  |  |  |  |  |  |  |  |  |
| Antihistamines | NR | NR | 64 (50.0) | 43 (43.0) | 56 (72.7) | 46 (59.7) | 38 (48.1) | 30 (50.8) | NR | 6 (17.1) | NR | NR | NR |
| Decongestants | NR | NR | 32 (25.0) | 6 (6.0) | 22 (28.6) | 12 (15.6) | 8 (10.1) | 5 (8.5) | NR | NR | NR | NR | NR |
| Leukotriene Inh. | NR | NR | 14 (10.9) | 4 (4.0) | 4 (5.2) | 5 (6.5) | 4 (5.1) | 2 (3.4) | NR | 1 (2.9) | NR | NR | NR |
| Intranasal Steroid | NR | NR | 82 (64.1) | 32 (32.0) | 34 (44.2) | 31 (40.3) | 28 (35.4) | 20 (33.9) | NR | 4 (11.4) | NR | NR | NR |
| Intranasal Antichol. | NR | NR | 33 (25.8) | 16 (16.0) | 19 (24.7) | 20 (26.0) | 14 (17.7) | 7 (11.9) | NR | 4 (11.4) | NR | NR | NR |
| Immunotherapy | NR | NR | NR | 3 (3.0) | NR | NR | 0 (0.0) | 0 (0.0) | NR | NR | NR | NR | NR |
| Others | NR | NR | NR | 4 (4.0) | NR | NR | 3 (3.8) | 2 (3.4) | NR | NR | NR | NR | NR |

**Table 2: Outcomes from the studies included.**

| Outcome Measure | Ehmer et al. Cohort | Lee et al. Cohort | RHINTRAC Cohort | CLARITY Cohort | Davis et al. Cohort | NEUROMARK |
| --- | --- | --- | --- | --- | --- | --- |
| Participants at Follow-up (n) |  |  |  |  |  |  |
| 3 months | NR | NR | 116 | 36 | NR | 125 |
| 6 months | NR | 129 | NR | NR | 80 | NR |
| 12 months | 50 | NR | 104 | 35 | NR | NR |
| 24 months | 34 | NR | 79 | NR | 75 | NR |
| 36 months | NR | 101 | 59 | NR | NR | NR |
| Total rTNSS |  |  |  |  |  |  |
| Baseline | 8.5 (95% CI 8.0, 9.0) | 7.8 (95% CI, 7.5–8.1) | 8.2 (95% CI, 7.9–8.6) | 7.78 ± 1.82 | 7.8 ± 1.8 | 7.8 (SD: 1.9) |
| 3 months | 3.4 (95% CI 2.8, 4.1) | 3.6 (95% CI, 3.2–4.0) | 4.6 (95% CI, 4.1–5.1) | 4.06 ± 2.24 | 3.7 ± 2.3 | 3.8 |
| 6 months | 3.3 ± 2.1 | 2.9 (95% CI, 2.5–3.3) | 4.0 (95% CI, 3.5–4.5) | NR | 3.5 ± 2.2 | NR |
| 12 months | 3.6 (95% CI 3.0, 4.3) | 2.8 (95% CI, 2.4–3.2) | 3.4 (95% CI, 2.9–3.9) | 4.0 ± 2.1 | 3.6 ± 2.7 | NR |
| 24 months | 2.9 ± 2.4 | 3.3 (95% CI, 2.8–3.8) | 2.9 (95% CI, 2.4–3.5) | NR | 3.5 ± 2.7 | NR |
| 36 months | NR | 3.2 (95% CI, 2.8–3.7) | 3.5(95%CI,2.9–4.1) | NR | NR | NR |
| Mean Change in rTNSS |  |  |  |  |  |  |
| 3 months | NR | NR | −3.6 (95% CI −4.2 to −3.0) | −3.72 ± 2.92 | NR | −4.0 (95% CI −4.6 to −3.4) |
| 6 months | NR | −4.9 (95% CI −5.5 to −4.3) | NR | NR | −4.2 (95% CI −4.8 to −3.6) | NR |
| 12 months | −4.9 ± 2.28 (estimated) | NR | −4.8 (95% CI −5.5 to −4.1) | −3.9 ± 1.97 (estimated) | NR | NR |
| 24 months | − 5.5 (95% CI, − 6.4 to − 4.6) | NR | −5.3 (95% CI −5.8 to −4.8) | NR | NR | NR |
| 36 months | NR | −4.5 (95% CI −5.1 to −4.0) | −4.7 (95% CI −5.3 to −4.1) | NR | NR | NR |
| Responder Rates (≥30% response) |  |  |  |  |  |  |
| 3 months | NR | NR | 67.5% (95% CI 55.9–77.8%) | ≥30% response 78% | NR | 73.3% (95% CI 64.0–82.7%) |
| 6 months | NR | 83.5% (95% CI 75.8–89.0%); ≥1-point MCID 95.0% (95% CI 89.6–97.7%) | NR | NR | 77.2% (95% CI 66.4–85.9%); ≥1-point MCID 91.1% (95% CI 82.6–96.4%) | NR |
| 12 months | 80.9% (95% CI 67.5–89.6%); ≥1-point response 100% (95% CI 92.4–100%) | NR | 80.6% (95% CI 69.1–89.2%) | ≥1-point MCID 91% (31/35); ≥30% response 80% (28/35) | NR | NR |
| 24 months | 88.2% (95% CI, 73.4%-95.3%) | NR | 87.3% (95% CI 78.0–93.8%) | NR | At both 6 and 24 months, ≥1-point MCID 91% | NR |
| 36 months | NR | 82.2% [95% CI: 73.6%−88.4%]) | 79.7% (95% CI, 67.2%–89.0%) | NR | NR | NR |
| Change in Congestion |  |  |  |  |  |  |
| 3 months | NR | NR | -1.0 (95% -1.2 to -0.8) | −1.11 ± 0.85 | NR | −1.0 (95% CI −1.2 to −0.8) |
| 6 months | NR | NR | NR | NR | NR | NR |
| 12 months | Mean score: 1.1 (95% CI ≈ 0.7 to 1.4) | NR | NR | NR | NR | NR |
| 24 months | Mean score: 0.9 (95% CI ≈ 0.6 to 1.2) | NR | NR | NR | NR | NR |
| 36 months | NR | NR |  | NR | NR | NR |
| Change in Rhinorrhoea |  |  |  |  |  |  |
| 3 months | NR | NR | -1.1 (95% -1.3 to -0.9) | −1.17 ± 1.00 | NR | −1.2 (95% CI −1.4 to −1.0) |
| 6 months | NR | NR | NR | NR | NR | NR |
| 12 months | Mean score: 1.2 (95% CI ≈ 0.8 to 1.5) | NR | NR | NR | NR | NR |
| 24 months | Mean score: 1.15 (95% CI ≈ 0.8 to 1.5) | NR | NR | NR | NR | NR |
| 36 months | NR | NR |  | NR | NR | NR |
| Change in Itching |  |  |  |  |  |  |
| 3 months | NR | NR | -0.8 (-1.0 to 0.5) | −0.78 ± 1.10 | NR | −0.9 (95% CI −1.1 to −0.7) |
| 6 months | NR | NR | NR | NR | NR | NR |
| 12 months | Mean score: 0.45 (95% CI ≈ 0.2 to 0.65) | NR | NR | NR | NR | NR |
| 24 months | Mean score: 0.4 (95% CI ≈ 0.15 to 0.6) | NR | NR | NR | NR | NR |
| 36 months | NR | NR | NR | NR | NR | NR |
| Change in Postnasal Drip |  |  |  |  |  |  |
| 3 months | NR | NR | NR | NR | NR | −1.1 (95% CI −1.4 to −0.9) |
| 6 months | NR | NR | NR | NR | −1.0 | NR |
| 12 months | Mean score: 1.9 (95% CI ≈ 1.4 to 2.4) | NR | NR | NR | NR | NR |
| 24 months | Mean score (2.1 (95% CI, 1.7 to 2.5) | NR | NR | NR | NR | NR |
| 36 months | NR | NR | NR | NR | NR | NR |
| Change in Sneezing |  |  |  |  |  |  |
| 3 months | NR | NR | -0.8 (-1.0 to -0.5) | −0.67 ± 1.01 | NR | −0.9 (95% CI −1.1 to −0.7) |
| 6 months | NR | NR | NR | NR | NR | NR |
| 12 months | Mean score: 0.75 (95% CI ≈ 0.4 to 1.0) | NR | NR | NR | NR | NR |
| 24 months | Mean score: 0.4 (95% CI ≈ 0.2 to 0.6) | NR | NR | NR | NR | NR |
| 36 months | NR | NR | NR | NR | NR | NR |
| Change in Cough |  |  |  |  |  |  |
| 3 months | NR | NR | NR | NR | NR | −0.9 (95% CI −1.1 to −0.6) |
| 6 months | NR | NR | NR | NR | −0.8 | NR |
| 12 months | Mean score: 1.0 (95% CI ≈ 0.6 to 1.4) | NR | NR | NR | NR | NR |
| 24 months | Mean score: 0.9 (95% CI, 0.5 to 1.3) | NR | NR | NR | NR | NR |
| 36 months | NR | NR | NR | NR | NR | NR |
| Change in MiniRQLQ |  |  |  |  |  |  |
| 3 months | NR | NR | NR | MiniRQLQ improved; MCID ≥0.4 in 89% | NR | MiniRQLQ −1.7 (95% CI −2.0 to −1.4) |
| 6 months | NR | -1.8 (-2.1 to -1.5) | NR | NR | −1.8 ± 1.3 | NR |
| 12 months | NR | NR | NR | MiniRQLQ 1.3 ± 0.8 | NR | NR |
| 24 months | NR | NR | −1.9 (95% CI, −2.1 to −1.7) | NR | −1.7 | NR |
| 36 months | NR | −1.8 (95% CI: −2.0 to −1.5; | −1.8 (95% CI −2.0 to −1.5) | NR | NR | NR |
| Depression Score |  |  |  |  |  |  |
| 3 months | NR | NR | NR | NR | NR | PHQ-9 −3.0 (95% CI −4.0 to −2.0) |
| 6 months | NR | NR | NR | NR | NR | NR |
| 12 months | NR | NR | NR | NR | NR | NR |
| 24 months | NR | NR | NR | NR | NR | NR |
| 36 months | NR | NR | NR | NR | NR | NR |
| Using Medications at Follow-up, n (%) |  |  |  |  |  |  |
| 3 months | NR | NR | NR | NR | NR | NR |
| 6 months | NR | NR | NR | NR | NR | NR |
| 12 months | More patients decreased antihistamine/decongestant, decongestant nasal spray, and steroid nasal spray use than increased use | NR | NR | NR | NR | NR |
| 24 months | NR | NR | Medication burden reduced | NR | NR | NR |
| 36 months | NR | Medication burden decreased | NR | NR | NR | NR |
| NOSE Score Change |  |  |  |  |  |  |
| 3 months | NR | NR | NR | NR | NR | −32.2 (95% CI −38.3 to −26.1) |
| 6 months | NR | NR | NR | NR | −33.5 (−39.3 to −27.7) | NR |
| 12 months | NR | NR | NR | NR | NR | NR |
| 24 months | NR | NR | NR | NR | −31.7 | NR |
| 36 months | NR | NR | NR | NR | NR | NR |
| Mean Nasal Pain |  |  |  |  |  |  |
| 3 months | NR | NR | 0.6 (95% CI, 0.2-0.9) | NR | NR | NR |
| 6 months | NR | NR | NR | NR | NR | NR |
| 12 months | NR | NR | NR | NR | NR | NR |
| 24 months | NR | NR | NR | NR | NR | NR |
| 36 months | NR | NR | NR | NR | NR | NR |
| Adverse Events, n (%) |  |  |  |  |  |  |
| 3 months | NR | NR | 3 device/procedure-related AEs, all resolved | No serious AEs | NR | 1 active sinusitis, 1 active orthostatic/presyncopal event, 1 sham sinus infection; all resolved |
| 6 months | NR | No serious device/procedure-related AEs through 6 months | NR | NR | 1 serious AE (epistaxis requiring hospitalization/SPA ligation); 9 nonserious related AEs in 8 participants | NR |
| 12 months | 16 device/procedure-related AEs in 8 patients across study; no serious related AEs | NR | No serious AEs reported in accessible text | No related serious AEs | NR | NR |
| 24 months |  | NR | No serious device/procedure-related AEs over 2 years | NR | No related serious AEs reported between 9 and 24 months | NR |
| 36 months | NR | No serious device- or procedure-related AEs | No severe AEs; no AEs between 24 and 36 months | NR | NR | NR |
| Other Outcomes |  |  |  |  |  |  |
| 3 months | NR | NR | Sham-controlled efficacy shown | Mean percent change in VAS rhinorrhea 53% and congestion 55% | NR | NOSE change: active −32.2 vs sham −8.0 |
| 6 months | NR | Satisfaction and recommendation outcomes also recorded | NR | NR | ETDQ-7 −1.2 | NR |
| 12 months | NR | NR | 57.8% improvement reported | 12-month observed values reported | NR | NR |
| 24 months | NR | NR | Combined index active + crossover active cohort | NR | ETDQ-7 −1.1 | NR |
| 36 months | NR | Durable 3-year improvement reported | Combined RHINTRAC active + crossover cohort | NR | NR | NR |
